# Characterization of Influenza Vaccine Immunogenicity Using Influenza Antigen Microarrays

**DOI:** 10.1371/journal.pone.0064555

**Published:** 2013-05-29

**Authors:** Jordan V. Price, Justin A. Jarrell, David Furman, Nicole H. Kattah, Evan Newell, Cornelia L. Dekker, Mark M. Davis, Paul J. Utz

**Affiliations:** 1 Department of Medicine, Division of Immunology and Rheumatology, Stanford University School of Medicine, Stanford, California, United States of America; 2 Department of Microbiology and Immunology, Stanford University School of Medicine, Stanford, California, United States of America; 3 Department of Pediatrics, Stanford University School of Medicine, Stanford, California, United States of America; 4 Stanford’s Institute for Immunity, Transplantation and Infection, Stanford, California, United States of America; University of Melbourne, Australia

## Abstract

**Background:**

Existing methods to measure influenza vaccine immunogenicity prohibit detailed analysis of epitope determinants recognized by immunoglobulins. The development of highly multiplex proteomics platforms capable of capturing a high level of antibody binding information will enable researchers and clinicians to generate rapid and meaningful readouts of influenza-specific antibody reactivity.

**Methods:**

We developed influenza hemagglutinin (HA) whole-protein and peptide microarrays and validated that the arrays allow detection of specific antibody reactivity across a broad dynamic range using commercially available antibodies targeted to linear and conformational HA epitopes. We derived serum from blood draws taken from 76 young and elderly subjects immediately before and 28±7 days post-vaccination with the 2008/2009 trivalent influenza vaccine and determined the antibody reactivity of these sera to influenza array antigens.

**Results:**

Using linear regression and correcting for multiple hypothesis testing by the Benjamini and Hochberg method of permutations over 1000 resamplings, we identified antibody reactivity to influenza whole-protein and peptide array features that correlated significantly with age, H1N1, and B-strain post-vaccine titer as assessed through a standard microneutralization assay (p<0.05, *q* <0.2). Notably, we identified several peptide epitopes that were inversely correlated with regard to age and seasonal H1N1 and B-strain neutralization titer (p<0.05, *q* <0.2), implicating reactivity to these epitopes in age-related defects in response to H1N1 influenza. We also employed multivariate linear regression with cross-validation to build models based on age and pre-vaccine peptide reactivity that predicted vaccine-induced neutralization of seasonal H1N1 and H3N2 influenza strains with a high level of accuracy (84.7% and 74.0%, respectively).

**Conclusion:**

Our methods provide powerful tools for rapid and accurate measurement of broad antibody-based immune responses to influenza, and may be useful in measuring response to other vaccines and infectious agents.

## Introduction

Each year, vaccines are generated against the most virulent strains of influenza to minimize global rates of morbidity and mortality associated with influenza infection [1]. Response to influenza vaccine varies greatly across the population, with notable deficits in vaccine response observed among the elderly [2]. Recent studies have shown that humans generate a remarkably broad immune response to influenza infection and vaccination, and that upon subsequent challenge with novel strains, preexisting influenza-specific B and T cell reactivity can have a positive or negative effect on an individual’s ability to neutralize the virus [3], [4], [5], [6]. Given the great heterogeneity of the human immune response to influenza infection and influenza vaccination, there exists a challenge and an opportunity to develop new approaches to better understand broad influenza-specific immunological responses [7], [8], [9]. Widely used methods to measure the effectiveness of influenza vaccine on induction of the humoral immune response include the hemagglutination inhibition (HAI) assay and the virus replication neutralization (microneutralization) assay, which indirectly measure the effect of vaccination on antibody reactivity [10]. While these assays are currently the gold standard for measuring antibody responses to influenza, and can, to an extent, predict protection from disease, they are limited in that each is specific for only a single antigen, and neither permits broad analysis of more limited epitope determinants recognized by immunoglobulins. Accordingly, proteomics platforms that can measure a great diversity of antibody binding to influenza antigen targets will be of great use for research and in the clinic for better understanding the antibody response to influenza virus and vaccine. To this end, we have developed influenza whole-protein and peptide antigen microarray platforms for the determination of antibody reactivity to conformational and linear epitopes of influenza hemagglutinin (HA). Other groups have reported array-based systems to measure the reactivity of a variety of HA-binding antibodies, including arrays generated with random peptides that can be used to measure the antibody response to influenza vaccination [11], [12], [13]. These studies demonstrate the potential value of an array-based approach. Arrays offer the advantage of a relatively straightforward measurement of antibody binding to protein and peptide features, require minimal blood sample volume (on the order of 2–15 µl of serum or plasma per measurement), do not rely on a complex biological setup (i.e., cell culture), and results can be obtained in a matter of hours. Whole-protein and peptide antigen arrays have been instrumental in defining epitopes for diverse protein interactions, including human patient autoantibodies [14], [15], [16], transplant-associated immunoglobulins [17], [18], antibodies generated by vaccination [19], chromatin-targeted antibodies [20], and substrates for kinase and methyltransferase enzyme activity [21], [22].

The goal of this study was to test whether array-based measurement can detect HA reactivity, specifically linear epitopes within HA, associated with effective or ineffective responses to influenza vaccination, as measured by the microneutralization titer in a population of young and older subjects. We analyzed serum from a cohort of individuals before and after vaccination with the 2008/2009 Fluzone™ seasonal trivalent influenza vaccine (TIV) on arrays containing viral proteins and HA peptides derived from seasonal H1N1, H3N2 and B influenza strains included in the TIV. Our analysis revealed whole protein and peptide reactivity that correlated positively and negatively with age and neutralization titer response to vaccine strains. Notably, we successfully predicted neutralization outcome for H1N1 and H3N2 influenza strains based on an individual’s age and pre-vaccine peptide reactivity with a higher level of accuracy than based on age alone.

The results of our study indicate that an array-based approach to survey the influenza-specific antibody repertoire is useful for defining meaningful epitope targets and can be used to predict the outcome of vaccination in individuals of varying ages. Ultimately, a better understanding of the humoral response to influenza vaccination and natural infection empowered by this technology, in combination with other approaches, may aid novel strategies to predict vaccine efficacy across populations.

## Materials and Methods

### Ethics Statement

A protocol for the study of healthy volunteers, ages 18 to ≥89 years, enrolled in an influenza vaccine study at the Stanford-Lucille Packard Children’s Hospital Vaccine Program, was approved in the summer of 2008 by the Institutional Review Board of the Research Compliance Office at Stanford University. Written informed consent was obtained from all subjects in the study.

### Subjects and Sample Collection

All individuals were ambulatory and generally healthy as determined by clinical history. Females of childbearing potential were tested for pregnancy by a urine sample. Volunteers at initial enrollment had no active systemic or serious concurrent illness, no history of immunodeficiency, nor any known or suspected impairment of immunologic function, including clinically significant liver disease, diabetes mellitus treated with insulin, moderate to severe renal disease, blood pressure >150/95 at screening, chronic hepatitis B or C, recent or current use of systemic immunosuppressive medication. In addition, none of the volunteers were recipients of blood or blood products within the past 6 months or blood donors within 6 weeks prior to immunization. Volunteers were screened to assure that they did not have signs of febrile illness on day of enrollment and baseline blood draw. A total of ∼120 ml peripheral blood was obtained in three visits (∼40 ml/visit): at day 0 (pre-vaccine), 5–7 and 28±7 days after receiving a single intramuscular dose of trivalent seasonal influenza vaccine Fluzone (Sanofi Pasteur, Swiftwater, PA). Each dose of the vaccine contained a total of 45 µg of antigen corresponding to 15 µg HA each of H1N1, H3N2 and B strains of the seasonal viruses included for that year. Serum was separated by centrifugation of clotted blood, and stored at −80°C before use. Serum samples from visit 1 (day 0) and 3 (day 28±7) were used for array experiments and microneutralization titer determination.

### Array Design and Fabrication

#### Whole-protein microarrays

We printed whole-protein antigens on nitrocellulose-surface glass slides (Whatman, Piscataway, NJ) using a VersArray ChipWriter Compact robotic microarrayer and ChipWriter Pro software (BioRad, Hercules, CA) as described [14], [23] in replicates of three across a range of concentration in phosphate-buffered saline (PBS, Bio-Rad).

#### Peptide microarrays

We printed streptavidin-surface glass slides (Arrayit, Sunnyvale, CA) with biotinylated 19-mer peptides (Sigma Aldrich, St. Louis, MO) diluted to a concentration of 0.5 mg ml^−1^ in peptide printing buffer [PBS plus 2.5% glycerol (Sigma)] in replicates of three using a VersArray ChipWriter Compact robotic microarrayer and ChipWriter Pro software (BioRad).

### Antibody-binding Assays

We blocked whole-protein arrays with 5% w/v non-fat milk (BioRad) in PBS for 1.25 h at room temperature (RT) with light rocking agitation. After rinsing arrays three times with whole-protein binding buffer (WPBB) [PBS plus 0.25% tween-20 (Sigma) (PBST) with 2.5% fetal calf serum (FCS, Omega Scientific, Tarzana, CA)], we applied indicated commercial mAb and pAb antibodies [HA-tag Ab, 14-6756-81 (eBioscience, San Diego, CA); FLAG-tag Ab, 200471 (Sigma); H1mAb, ab66189; H1pAb, ab91531; H3 mAb, ab66187 (Abcam, Cambridge, MA); H3 pAb, IA-PAN4-0100 (eEnzyme, Gaithersburg, MD) diluted to 1.0 µg ml^–1^ (or as indicated) or patient serum diluted 1∶200 in WPBB for 1.25 h at 4°C with light rocking agitation. We then rinsed the arrays three times followed by three 5 min washes in WPBB before applying secondary antibody reagents (goat anti-mouse IgG, goat anti-rabbit IgG, or goat anti-human IgG conjugated to Cy5, Jackson ImmunoResearch, West Grove, PA) diluted to 0.375 µg ml^–1^ in WPBB. Following incubation of detection antibody for 45 min at 4°C with light rocking agitation, we rinsed arrays three times followed by three 5 min washes in WPBB, then rinsed arrays in PBS followed by diH_2_0. We dried arrays in microscope slide racks centrifuged at 300 *g* for 5 min at RT.

We blocked printed peptide arrays with 0.1 mg ml^−1^ biotin (Sigma) in PBS for 10 min at RT with 500 rpm orbital agitation. After rinsing the arrays six times in peptide binding buffer (PBB): [50 mM Tris (Mallinckrodt Baker, Phillipsburg, NJ) pH 7.5, 150 mM NaCl (Mallinckrodt AR, Phillipsburg NJ), 0.05% NP-40 (Sigma) +2.5% FCS), we applied mAb and pAb antibodies diluted to 1.0 µg ml^–1^ or patient serum diluted 1∶80 in PBB. Following primary incubation for 3 h at RT with 500 rpm orbital agitation, we rinsed the arrays six times with PBB then six times in PBST before applying secondary antibody reagents (goat anti-mouse IgG, goat anti-rabbit IgG or goat anti-human IgG conjugated to Cy3, Jackson ImmunoResearch) diluted to 0.375 µg ml^–1^ in PBST plus 20% FCS for 30 min at RT with 500 rpm orbital agitation. We then rinsed arrays six times in PBST, three times in PBS and three times in diH_2_0 and dried arrays in microscope slide racks centrifuged at 300 *g* for 5 min at RT. We immediately scanned processed whole-protein and peptide arrays with an Axon digital scanner and analyzed scanned images with Genepix Pro 6.1 software (Molecular Devices, Sunnyvale, CA).

### Pre-clearing Assay

For pre-clearing antigen-specific antibodies in array and ELISA experiments, we incubated streptavidin-sepharose beads (GE Healthcare, Piscataway, NJ) with the indicated biotinylated peptides diluted to 0.2 mg ml^–1^ in PBS for 30 min at RT, followed by two washes with PBS +10% FCS. We then incubated peptide-bound beads with the indicated antibodies diluted to 6.0 µg ml^–1^ in PBB +2.5% FCS for 20 min at RT. Following incubation, we centrifuged the mixture at 850 *g* for 1 min to pellet the bead/Ab complexes. We repeated the pre-clearing procedure three times, at each stage reserving part of the pre-cleared reactant for probing arrays and ELISA.

### ELISA

We coated Nunc-Immuno Maxisorp 96-well plates (Thermo Scientific, Rochester NY) with the indicated peptides diluted to 1.0 µg ml^−1^ in carbonate buffer, pH 9.5≥12 h at 4°C. We rinsed the plates three times with PBST and blocked with PBST +10% FCS for 30 min at RT. Following the blocking step, we rinsed the plates three times with PBST and applied probing reagents (mAb/pAb commercial antibodies or patient serum) diluted in PBST plus 10% FCS in triplicate wells (50 µl/well), and incubated 2 h at RT. We then rinsed the plates three times with PBST, applied 100 µl/well PBST +10% FCS and gently vortexed the plates before washing three times with PBST. We then incubated peroxidase–conjugated goat anti-human IgG (H+L) for human serum samples, or peroxidase-conjugated donkey anti-mouse IgG (H+L) secondary antibodies (Jackson ImmunoResearch) for mouse primary antibodies at 0.16 µg ml^–1^ in PBST +10% FCS on the plates for 1 h at RT. We washed the plates as described following the primary incubation step, followed by detection with TMB One-Step Substrate (Dako, Carpinteria, CA). We terminated the TMB reaction with 2 M sulfuric acid (Sigma) and obtained colorimetric readings with a SpectraMAX 190 plate reader at λ = 450 (Molecular Devices, Sunnyvale, CA).

### Influenza Microneutralization Assay

Influenza virus microneutralization assay was performed according to standard procedure [10]. Briefly, the 50% tissue culture infective dose (TCID_50_) for H1N1 (A/South Dakota/6/2007), H3N2 (A/Uruguay/716/2007) and B (B/Florida/4/2006-like) influenza virus strains (kindly provided by MedImmune, Santa Clara, CA) was determined in Madin-Darby Canine Kidney-London (MDCK-London) cells (gift from Dr. David Lewis, Stanford University) cultured at a density of 1.5×10^5^ cells/well in a 96-well microtiter plate (Immulon2 HB, Thermo Scientific, Rochester, NY). Titration of each human serum was performed in duplicate. Sera were heat inactivated for 30 minutes at 56°C, serially diluted from 1∶10 to 1∶1280, and incubated with 100 TCID_50_ virus/well for 1 hr at 37°C before being applied to 1.5×10^5^ MDCK cells for 18–22 hours at 37°C. Following fixation with cold 80% acetone (Fisher Scientific, Pittsburgh, PA) in PBS, the MDCK monolayer was probed in an ELISA assay, first with a 1∶1 mixture of MAB8257 and MAB8258 (Millipore, Billerica, MA), followed by peroxidase-conjugated goat anti-mouse IgG (Kirkegaard and Perry Laboratories, Gaithersburg, MD). Optical density following development with o-phenylenediamine dihydrochloride substrate (Sigma) was used to calculate the final neutralization titer for each serum.

### Statistical Analysis

For correlation of reactivity to individual peptide and whole-protein array features with age or cPost titer, we used a logarithmic transform of array feature MFI (log_10_ MFI) and normalized reactivity corresponding to arrays incubated with pre- and post-vaccine samples by subtracting the logarithmic transform of the local background MFI at each feature. The inverse log of the resulting value for each feature (log_10_ feature MFI minus log_10_ local background MFI) was used for correlation and multivariate prediction analyses. Post-vaccine neutralization titer was corrected for the influence of pre-existing antibody reactivity as described [24], [25]. Briefly, the logarithmic transform of post-vaccine titer for each patient was adjusted based on the slope of a linear regression of pre- and post-vaccine titer measurements for each influenza strain:
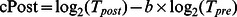
Where *T_post_* and *T_pre_* are traditional titer measurements (0–1280 corresponding to dilution factors of patient serum in which neutralization activity was observed) and *b* is the slope of a linear regression of pre-and post titer measurements for all samples tested. In a population partially seropositive before vaccine, this transformation of post-vaccine titer (cPost) corrects for the positive influence of pre-existing reactivity on post-vaccine titer. We used simple, least-square linear regression to calculate regression coefficient (β) and p value associated with individual peptide and whole protein array feature reactivity with age and cPost neutralization titer for H1N1, H3N2 and B strain influenza. False discovery rate (FDR, *q* value) was calculated using 1000 random permutations of the samples.

For multivariate prediction analysis, we set windows for good and poor response to vaccine as upper and lower quartile of cPost neutralization titer for each strain, respectively. We used age and pre-vaccine peptide feature MFI as predictors of high or low quartile cPost neutralization response. We used leave-one-out cross-validation for feature selection and estimation of prediction accuracies. We used the elastic net penalty [26] which combines both l1 and l2 penalties. The optimization cost can be stated as:
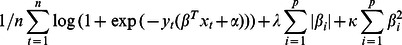
where *n* is the number of donors in the sample, *p* is the number of predictors, *x*_t_ denotes a vector of predictor values for subject *t* and *y_t_* is the observed outcome. We assume all of our predictors are standardized to mean 0 and standard deviation 1.

## Results

### Array Design and Fabrication

**Figure 1:**
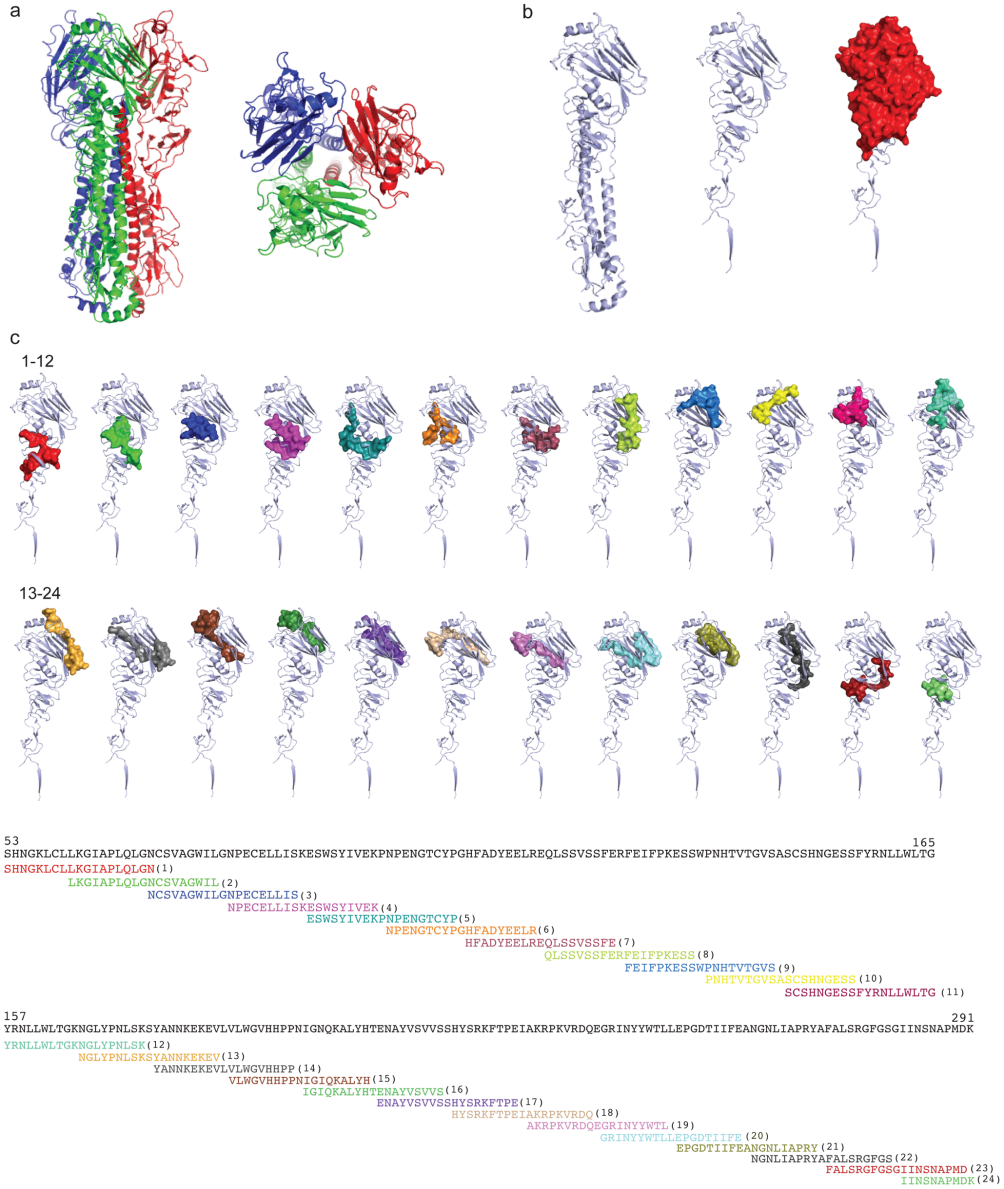
Hemagglutinin (HA) structure and synthetic peptides. (**a**) Side and top view of 3D structure of H1N1 A/Solomon Islands/3/2006 (Research Collaboratory for Structural Bioinformatics, Protein Data Bank PDB#3SM5) hemagglutinin (HA) trimer. (**b**) Individual HA monomer with head and stalk regions (left), head domain alone (center), and head domain showing the region spanning amino acids 53–291, highlighted in red (right), which we selected for synthesis of overlapping peptides. (**c**) Individual H1N1 (A/Brisbane/59/2007) peptides (1–24) are shown in the context of the head domain 3D structure (above) and amino acid sequence (below), with colors corresponding to each unique synthesized peptide. For depiction of peptides in **c**, we aligned the sequence of H1N1 A/Brisbane/59/2007 to H1N1 A/Solomon Islands/3/2006 and highlighted aligned homologous peptides. Seasonal H3N2 and B strain peptides are not depicted.

Influenza HA is a homotrimeric protein containing head and stalk regions that functionally interact with host cell receptors to facilitate entry of the virus into cells [27] (**Fig. 1a**). For our studies, we focused on a broad region of HA containing multiple known antibody epitopes important for neutralizing activity against influenza [28] (**Fig. 1b**). We synthesized 19-mer peptides overlapping by 10 amino acids that span the head region of H1N1 (A/Brisbane/59/2007), H3N2 (A/Brisbane/10/2007), and B (B/Brisbane/60/2008) influenza strains included in the 2008/2009 TIV as well as control peptides containing well-described HA-tag (NH_2_-YPYDVPDYA-COOH) and FLAG-tag (NH_2_-DYKDDDDK-COOH) sequences [29], [30] (**Fig. 1c** and **Table S1**). We incorporated a biotinylated aminohexanoic acid linker at the N-terminus of each peptide, which allowed us to immobilize and orient the peptides on streptavidin-coated glass slides in arrays containing 216 HA peptide features representing 72 unique peptides (**Fig. 1c** and **Table S1**). We adapted array-processing methods based on previously described protocols [15], [20] (**Fig. S1**). In parallel, we developed a nitrocellulose-based platform on which we printed whole-protein recombinant HA corresponding to 2008/2009 influenza A viral strains; inactivated seasonal H1N1 and H3N2 viruses; the 2008/2009 Fluzone™ TIV; alongside control proteins, including U1-A snRNP (spliceosome-associated protein) and separate measles, mumps and rubella control vaccine antigens (**Table S1**).

### Peptide Array Specificity, Sensitivity and Dynamic Range

**Figure 2:**
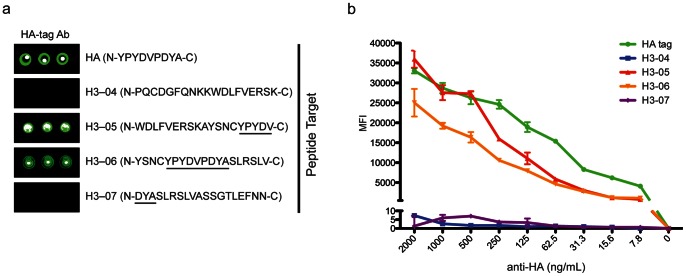
Specificity and broad dynamic range of peptide arrays. (**a**) Images displaying reactive peptide features from an array probed with HA-tag antibody (eBioscience 14-6756-81). Amino acid sequence of peptide targets is displayed in parentheses. Residues corresponding to HA-tag sequence (NH_2_-YPYDVPDYA-COOH) are underlined in seasonal H3N2 peptide sequences. (**b**) Graph displaying MFI of array peptide features depicted in **a** in a titration experiment using HA-tag antibody ranging in concentration from 2000 ng mL^−1^ to 0 ng mL^−1^. Error bars in **b** reflect mean ± SEM of triplicate array feature MFI for each titration point.

We first assessed specificity, sensitivity, and dynamic range of the peptide array platform using commercially available antibodies directed against HA-tag and FLAG-tag peptides. Polyclonal and monoclonal antibodies targeting HA-tag and FLAG-tag peptides bound to array features containing HA-tag and FLAG-tag epitope sequences, respectively, and did not cross react with each other (**Fig. S2a**). Within the sequence of seasonal H3N2 HA covered by the peptides we synthesized, the exact HA tag sequence is represented (amino acids 114 to 122 of H3N2 peptides, **Table S1**). We detected reactivity of the anti-HA-tag pAb only at overlapping H3N2 peptides on the array containing full or partial HA-tag sequence (**Fig. 2a**). Interestingly, we observed the highest level of binding of the anti-HA-tag pAb to HA peptide H3–5, a peptide containing only a partial overlap of the HA-tag sequence (**Fig. 2a** and **Table S1**). This indicates that only the 5-most N-terminal residues (NH_2_-YPYDV-COOH) of the HA-tag sequence are required for binding of this antibody, and that there may be orientation requirements for optimal recognition of the sequence within a larger peptide epitope. Titration of the anti-HA-tag pAb on the array reflected a linear range of detection across three logs of antibody concentration (**Fig. 2b**). ELISA results using the same reagents confirmed the specificity observed on the array (**Fig. S2b**). Direct comparison of the antibody titration showed that while signal was slightly higher when measured by ELISA vs. peptide microarray for these targets, the array signal more accurately demonstrated the linear change in concentration of antibody across the range of titration (data not shown).

**Figure 3:**
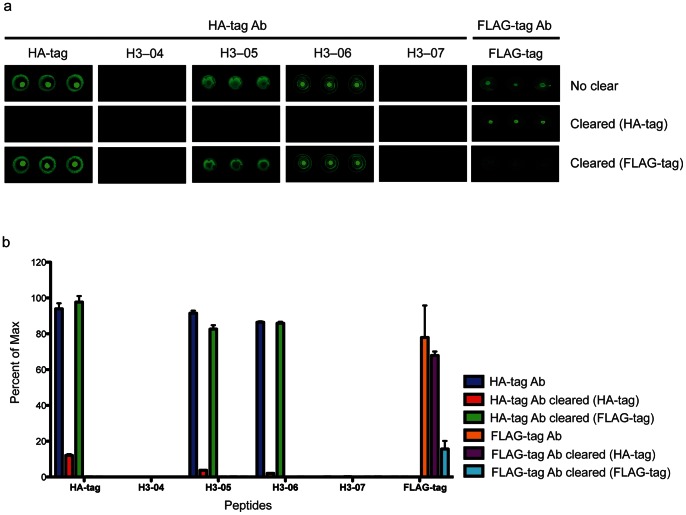
Peptide arrays allow for discrimination of unique peptide antibody targets. (**a**) Images displaying reactive HA-tag, seasonal H3N2 H3–4-7 and FLAG-tag peptide features on arrays probed with HA-tag antibody (14-6756-81, eBioscience) or FLAG-tag antibody (A9594, Sigma) either not pre-cleared, or after three rounds of clearing with HA-tag peptide or FLAG-tag peptide conjugated to streptavidin beads. (**b**) Histogram displaying percent (%) of maximum MFI for peptide features shown in **a**. Error bars in **b** represent the mean ± SEM of % of maximum MFI of triplicate array features. For each peptide, % of maximum MFI of the highest MFI replicate feature (of three) in the non-pre-cleared condition was calculated.

We further assessed the specificity of array peptide reactivity using HA-tag and FLAG-tag peptides conjugated to streptavidin-coated sepharose beads to deplete samples containing either anti-HA-tag or anti-FLAG-tag antibodies before probing arrays. We observed a significant decrease in reactivity to HA-tag peptide targets in the anti-HA-tag sera cleared with HA-tag peptide (**Fig. 3**). Conversely, the anti-HA-tag sera cleared with non-specific FLAG-tag peptide showed no reactivity difference compared to the same array features probed with nondepleted anti-HA-tag antibody (**Fig. 3**). In summary, our validation results indicate that peptide arrays can be used to resolve specific HA reactivity across a wide range of antibody concentration.

### Array Reactivity of Antibodies Generated against Conformational Targets

**Figure 4:**
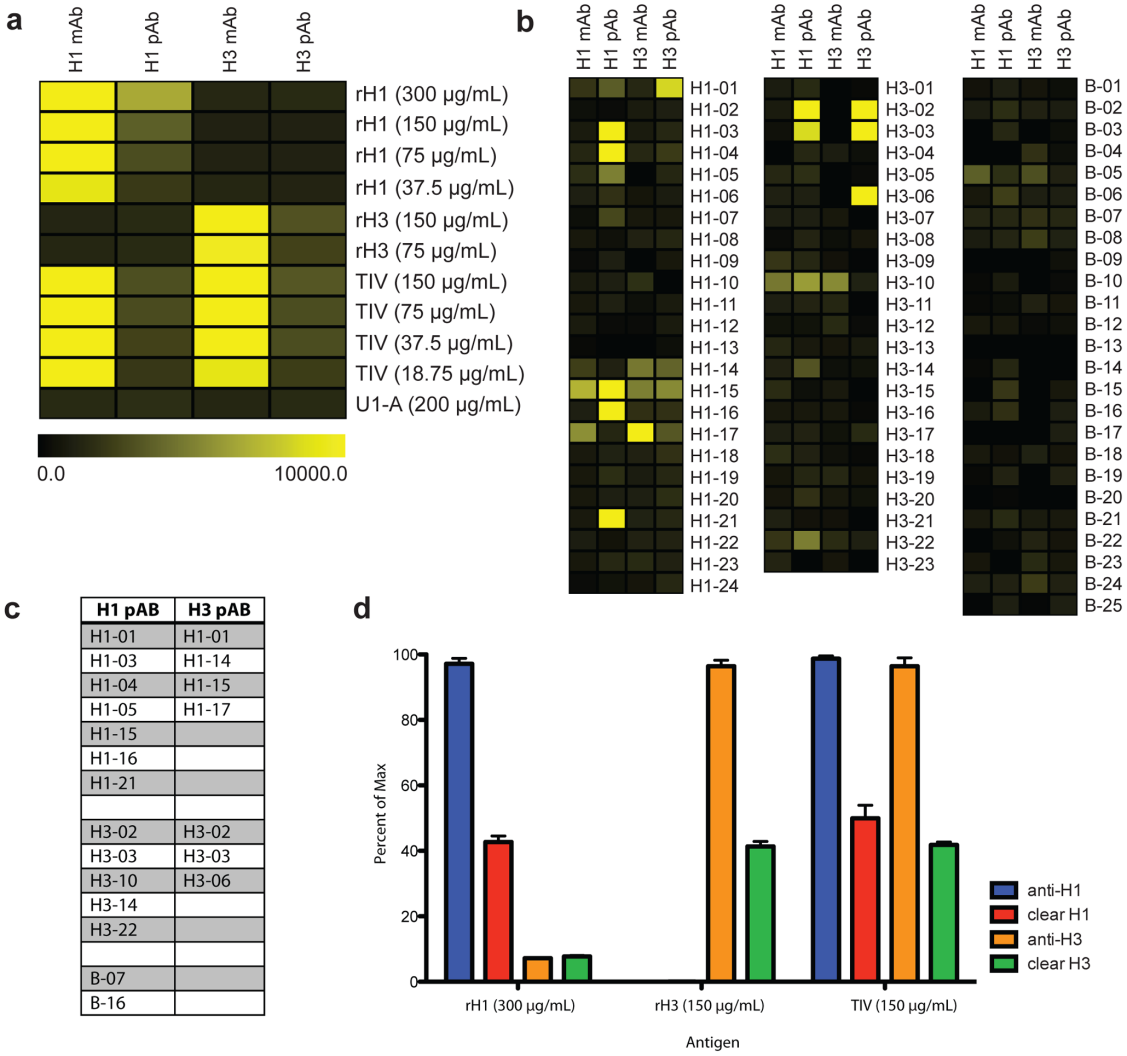
Decreased reactivity of commercial HA antibodies to recombinant HA proteins and influenza vaccine after pre-clearing with linear HA peptides. (a) Heat map displaying reactivity of commercial antibodies raised against purified virus or recombinant HA on a nitrocellulose array containing recombinant HA proteins, trivalent influenza vaccine (TIV – Fluzone seasonal 2008/2009 vaccine) and U1-A spliceosome protein printed at indicated concentration. Scale bar reflects median fluorescence intensity (MFI) of antibody-bound array features in a and b. H1mAb, ab66189; H1pAb, ab91531; H3 mAb, ab66187 (Abcam); H3 pAb, IA-PAN4-0100 (eEnzyme). (b) Heat maps displaying reactivity of indicated antibodies when incubated on the influenza peptide array. Antibodies are the same as those described in a. (c) Highest reactivity peptides bound by H1pAb and H3pAb. (d) Histogram displaying percent of maximum reactivity of HA antibodies H1pAb and H3pAb cleared and not cleared with selected peptides to recombinant H1, recombinant H3, and Fluzone vaccine antigens. Error bars represent the mean ± SEM of % of maximum MFI of triplicate array features. For each antigen, % of maximum MFI of the highest MFI replicate feature (of three) in the non-pre-cleared condition was calculated.

To test whether antibodies generated *in vivo* against influenza proteins will bind to whole-protein and peptide array targets we probed both whole-protein and peptide influenza arrays with commercial antibodies raised against purified H1N1 and H3N2 viruses or recombinant HA (rHA) in mouse and rabbit. As expected, these antibodies bound to cognate rHA antigens on the whole-protein array, as well as whole inactivated H3N2 and H1N1 seasonal viruses and the 2008/2009 TIV, but not to non-specific viral targets or U1-A control antigen (**Fig. 4a**). The mAb and pAb probes also displayed reactivity to a subset of H1N1, H3N2, and B strain peptide features on the HA peptide arrays, indicating that antibodies raised against whole-protein immunogens have the capacity to bind to linear peptide targets (**Fig. 4b**). To further demonstrate that linear HA epitopes on our platform are bound by antibodies raised against influenza proteins, we selected the H1 polyclonal antibody (Abcam) and the H3 polyclonal antibody (eEnzyme) and performed an antibody pre-clearing experiment with bead-bound HA peptides. Highest reactivity peptides bound by each pAb (**Fig. 4c**) were pooled and incubated with SA beads to make two unique pre-clear cocktails. Clearing of anti-H1N1 HA pAb and anti-H3N2 HA pAb with pooled peptide sets resulted in decreased reactivity to corresponding recombinant HA targets and to TIV antigens on the whole-protein influenza array (**Fig. 4d**). This result shows that antibodies raised against whole-protein influenza antigens react with influenza peptides, and supports the hypothesis that peptide arrays can be used for epitope mapping with antibodies raised against influenza HA *in vivo*.

**Figure 5:**
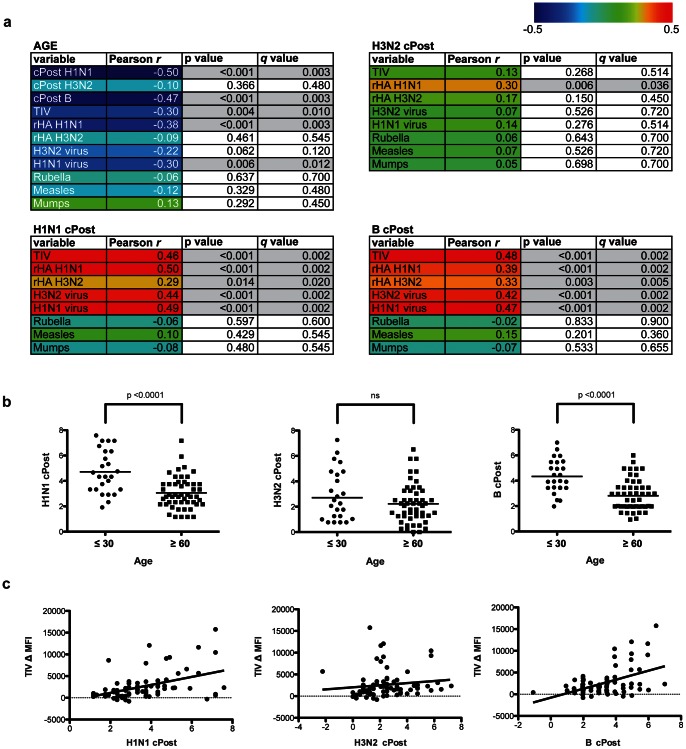
Correlation of age and post-vaccine neutralization titer with influenza whole-protein array reactivity. (**a**) Table displaying correlation coefficient (Pearson *r*), p value and false discovery rate (FDR) *q* value of whole-protein influenza antigen array reactivity with age and corrected post-vaccine neutralization titer (cPost) for H1N1, H3N2 and B influenza strains. Correlations with a p value <0.05 are highlighted in gray. Color scale represents the direction and magnitude of the Pearson *r* correlation coefficient. (**b**) Comparison of age and cPost H1N1, H3N2 and B influenza strain neutralization titer. P values were generated using a two-tailed student’s T test. (**c**) Dot plots displaying relationship between cPost neutralization titer (x axes) and change in array reactivity (MFI) to Fluzone trivalent influenza vaccine (TIV Δ, y axes). The line in each graph displays the least-squares linear regression of cPost titer and TIV Δ MFI, and corresponds to the Pearson *r* values shown in **a**.

To determine whether the arrays can be used to characterize human antibody responses to influenza antigens, we incubated our optimized and validated whole-protein and peptide arrays with individual serum samples derived from a cohort of 76 individuals in which samples were obtained immediately before and approximately 28 days following vaccination with the 2008/2009 Fluzone™ TIV (**Table S2**). We performed microneutralization assays [10] using seasonal H1N1, H3N2 and B viruses to determine the level of response to TIV as measured by antibody titer, with higher neutralization titer reflecting a stronger antibody response to the virus. An observed phenomenon in the immune response to influenza is the presence of pre-existing immunity that negatively correlates with increased response to vaccine and may actively suppress novel responses to vaccination [3], [6]. To account for this, pre- and post-vaccine titers were transformed into binary logarithms and corrected for pre-vaccination status as previously described [24], [25]. Corrected post-vaccine (cPost) neutralization titer for H1N1 and B strains was significantly negatively correlated with the age of study participants, a phenomenon that has been documented by our groups and others [31] (**Fig. 5a and 5b**). H3N2 cPost titer was not correlated with age, in agreement with observations of a more age-independent immune response to this strain of influenza [32], [33], [34] (**Fig. 5a and 5b**).

### Whole-protein Influenza Arrays Reveal Reactivity that Correlates with Neutralization Titer

To initially explore our ability to make and test hypotheses using our sample cohort, we ran pre- and post-vaccine samples on the whole-protein influenza array. We used a simple regression model of the change in MFI reactivity observed to whole-protein antigens (post minus pre, “Δ”) by age and cPost titer. To test the robustness of our associations and account for multiple hypothesis testing, we used the Benjamini and Hochberg method [35] of permutations over 1000 resamplings. In addition to H1N1 and B strain cPost titer, reactivity to H1N1 rHA and inactivated whole H1N1 virus, as well as TIV, but not H3N2 antigens or control antigens (rubella, measles and mumps antigens) was significantly negatively correlated with age (p<0.05, false discovery rate (FDR) *q* value <0.05) (**Fig. 5a**). The array did not contain B strain antigens, other than from those included within the TIV. This result demonstrates the robustness of our array-based method to obtain meaningful measurements of antibody reactivity to influenza antigens, and to draw biological connections between array reactivity, age and neutralization titer data associated with our sample cohort.

Post-vaccine change in reactivity to H1N1 and H3N2 rHA, inactivated whole viruses, and the TIV all correlated significantly with H1N1 and B cPost titer (p value <0.05, *q* value <0.05) (**Fig. 5a and 5c**). These results suggest that H1N1 and B strain-targeted antibodies, including those targeted specifically to influenza HA protein, are important for the ability to neutralize H1N1 and B strain viruses, and that antibodies neutralizing H1N1 or B strain influenza may cross-react with other strains. The phenomenon of HA-targeted antibodies that cross-react with different strains of influenza is well documented; specifically, H1N1 HA-targeted antibodies have been shown to cross-react with other strains of influenza in a neutralizing capacity [5], [36], [37]. Interestingly, cPost titer to H3N2 virus correlated with reactivity to H1N1 rHA but did not correlate significantly with change in H3N2 array antigens or TIV reactivity (**Fig. 5a**). This result reflects that *in vitro* neutralization of H3N2 virus may depend on antibodies targeted to H3N2 proteins or epitopes not contained on the whole-protein array, and that neutralizing H3N2-targeted antibodies may cross-react with H1N1 HA. In summary, these results confirm that our array-based approach allows for the measurement of meaningful influenza-specific antibody reactivity, and that array reactivity within our cohort reflects previously observed trends with respect to age and correlation with changes in post-vaccine neutralization titer.

### Peptide Array Antibody Reactivity Correlates Significantly with Age and Neutralization Titer

**Figure 6:**
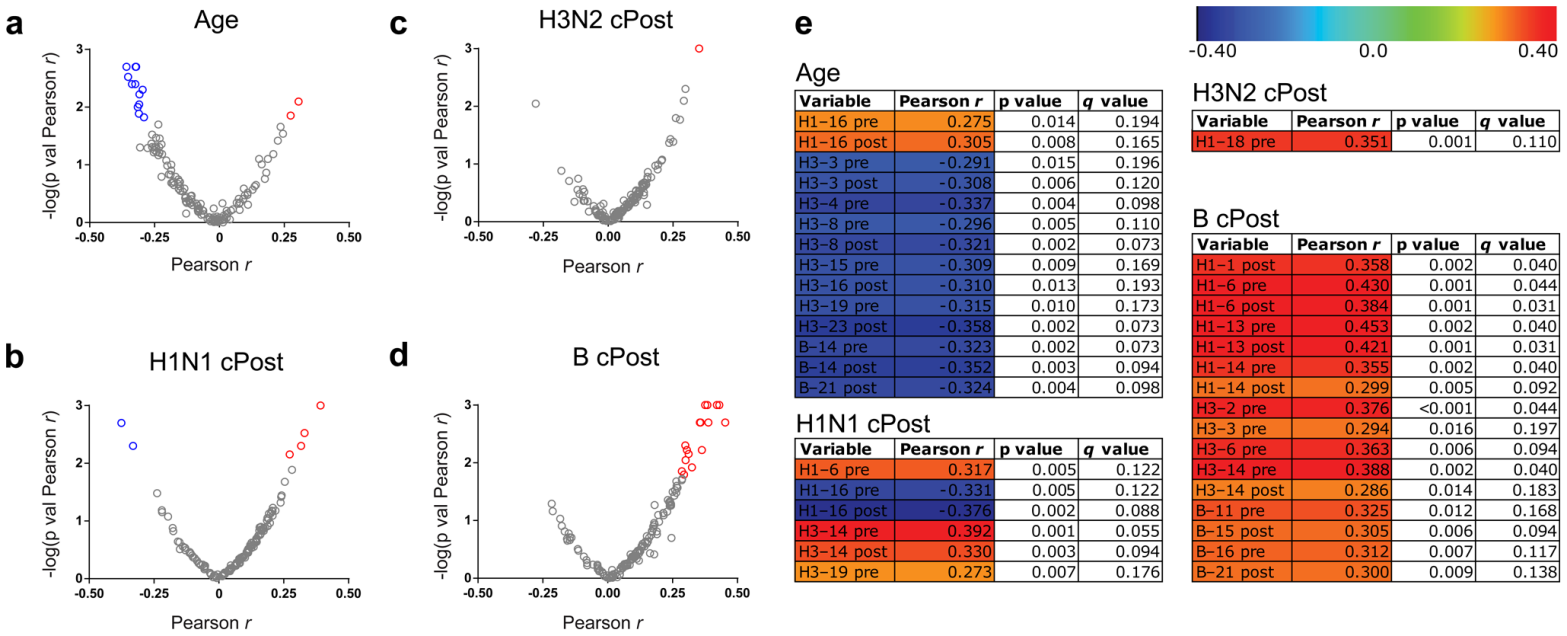
Influenza HA peptide reactivity correlates with age/post-vaccine neutralization titer. (**a–d**) Volcano plots displaying significance of the Pearson *r* correlation coefficient “−log(p value Pearson *r*)”, corresponding to the relationship of (**a**) age; (**b**) H1N1 cPost; (**c**) H3N2 cPost; and (**d**) B cPost neutralization titer with pre- and post-vaccine reactivity to H1N1, H3N2 and B-strain peptides. The Pearson *r* correlation coefficient is plotted on the x-axis. Red and blue color represents positive or negative correlation, respectively, of associations with a *q* value determined to be less than 0.2. Gray color indicates peptide associations that were not significant. (**e**) Table and heatmap displaying correlation coefficient (Pearson *r*), p value and false discovery rate (FDR) *q* value of the significant peptide reactivity shown in **a–d** with age, H1N1 cPost, H3N2 cPost, and B cPost. Color scale represents the direction and magnitude of the Pearson *r* correlation coefficient. See tables S3, S4, S5, S6, for the statistical results of all comparisons.

To test whether peptide array reactivity correlated with age and cPost titer, we incubated pre- and post-vaccine serum from the 76-person study cohort on the influenza HA peptide array. As was done for the whole-protein data, we performed simple regression over 1000 permutations of three measurements per peptide: pre-vaccine reactivity, post-vaccine reactivity, and the change in reactivity (post minus pre, “Δ”), by age or cPost titer for each influenza strain. Intriguingly, very few Δ variables were associated with age or cPost titer for each strain (data not shown). However, several pre- and post-vaccine peptide variables correlated significantly with age (p value <0.05, *q* value <0.2) (**Fig. 6a, 6e** and **Table S3**). We also observed peptide variables that correlated significantly with H1N1, H3N2 and B cPost titer (p value <0.05, *q* value <0.2) (**Fig. 6b–e** and **Tables S4, S5, S6**). Analysis of the peptide-binding data led us to make several observations. The location of reactive peptides that significantly correlated with age and cPost neutralization titer reveals considerable overlap with the region of HA known to contact its host receptor, sialic acid, indicating that antibody targeting of these epitopes may be critical for effective neutralization of influenza host cell entry [28], [38], [39] (**Fig. S3**). As with the neutralization titer data and whole-protein reactivity, most of the peptide reactivity correlations with age were negative, indicating that the peptide array allowed us to identify specific linear epitopes within seasonal H1N1, H3N2 and B-strain hemagglutinin that are not effectively targeted in elderly individuals (**Fig. 6a** and **Table S3**). Conversely, most peptide reactivity correlations with H1N1, H3N2 and B cPost titer were positive, identifying potential target regions of HA for effective neutralizing antibodies (**Fig. 6b–d** and **Tables S4, S5, S6**). Several of the same peptides that were negatively correlated with age were positively correlated with H1N1 and B-strain neutralization titer, delineating specific epitopes within HA potentially important for effective response to these strains deficient in the elderly (**Fig. 6**, **Fig. S3**, and **Tables S4, S5, S6**). Of particular note, we also observed that both pre- and post-vaccine reactivity to the H1–16 peptide, spanning the sequence NH_2_–IGIQKALYHTENAYVSVVS–COOH was positively correlated with age and negatively correlated with H1N1 neutralization titer (**Fig. 6a, 6b**, **Fig. S3**, and **Tables S4**). Interestingly, this peptide contains partial overlap with a known T cell and B cell epitope within the receptor-binding domain of HA; however, other investigators observed that antisera raised against a peptide derived from this region did not have neutralizing capacity [40], [41], [42]. This raises the possibility that antibody targeting of the epitope contained within H1–16 may play a role in age-related deficiency in response to H1N1. The peptide analysis also allowed us to identify regions of potential antibody cross-reactivity between strains, as cPost titer for each strain tested was associated with reactivity to peptides from at least one different strain (**Fig. 6b–d** and **Tables S4, S5, S6**). Specifically, we found that reactivity to two H3N2 peptides in addition to two H1N1 peptides was associated with H1N1 neutralization response to vaccine, and that reactivity to peptides from three influenza strains correlated with B strain neutralization response (**Fig. 6b and 6d**, **Fig. S3** and **Tables S4** and **S6**). These results suggest that cross-reactivity induced by vaccination may be relevant to neutralization of different influenza strains, and that this reactivity can be mapped, at least partially, using HA peptide arrays.

**Figure 7:**
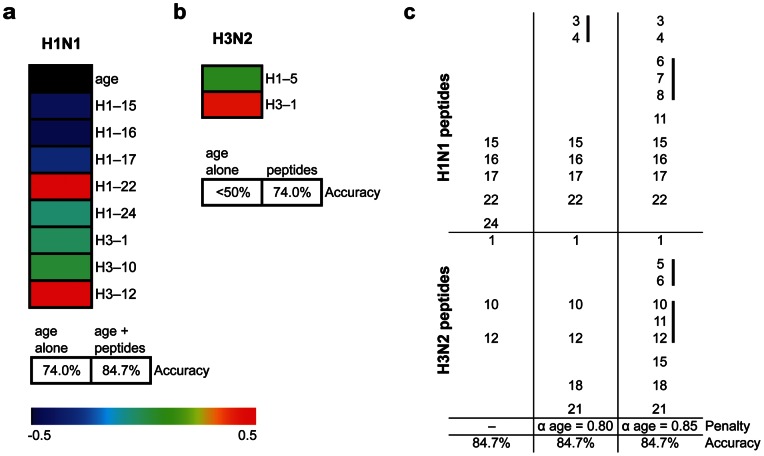
Analysis of age and pre-vaccine peptide reactivity can predict effective neutralization outcome. (**a,b**) Tables showing peptides selected in a multivariate prediction model that identified vaccine recipients as upper or lower quartile responders with respect to H1N1 (**a**) and H3N2 (**b**) cPost neutralization titer verified using leave-one-out (LOO) cross-validation. The accuracy of using age alone or the model presented in **a** and **b** for prediction of good (upper quartile cPost titer) or poor (lower quartile cPost titer) response to vaccine is shown below. Color scale indicates direction and magnitude of the regression coefficient (β) for each element in **a** and **b**. (**c**) Peptides selected in multivariate prediction models of H1N1 cPost-vaccine titer with no age penalty, or increasing age penalty (increasing α age value) using the elastic net method (see methods). Black bars indicate adjacent peptides (overlapping sequence) selected in models using age penalty.

We also observed that a number of pre-vaccine samples displayed reactivity that correlated significantly with cPost titer of all strains (p value <0.05, *q* value <0.2) (**Fig. 6b–d** and **Tables S4, S5, S6**). This observation raised the possibility that the assessment of pre-vaccine peptide reactivity may be sufficient to determine the outcome of antibody response to the vaccine, as measured by neutralization titer. To test this, we selected study participants at the extremes of the cPost titer distribution for each strain (46/76 for H1N1; 49/76 for H3N2; and 46/76 for B) and classified them as good responders (upper quartile) and poor responders (lower quartile). We applied multivariate regression analysis using age and pre-vaccine peptide reactivity to build prediction models capable of classifying participants as falling into good or poor responder categories. We employed leave-one-out (LOO) cross-validation using the elastic net algorithm [26] to computationally select peptide variables that, jointly, could prospectively determine the neutralization response and estimate the performance of our prediction model. In these cross-validation processes, we divided the samples into *n* (*n* = number of samples included in the analysis) equal partitions, and applied a regression model to all but one partition, which served as training set. The remaining partition served as the validation/test set for the model. We iterated this process *n* times and assessed the performance of the model by computing the misclassification error in the excluded partition. For H1N1, our prediction model included age and eight pre-vaccine peptide variables, and predicted H1N1 cPost titer outcome with 84.7% accuracy (**Fig. 7a**). This model reflects an improvement of >10% above prediction using age alone (**Fig. 7a**). For H3N2, our prediction model incorporated pre-vaccine reactivity to two array peptides, and predicted cPost titer response with 74.0% accuracy (**Fig. 7b**). For H3N2, age does not predict at a level better than 50% (random classification) (**Fig. 7b**). The prediction of cPost titer outcome for B strain influenza based on pre-vaccine peptide reactivity did not yield results with accuracy above 50% (random classification, data not shown). These results indicate that measurement of pre-vaccine reactivity to peptide targets can improve upon prediction of response based solely on age for H1N1, and constitute an age-independent method to accurately predict H3N2 neutralization response to vaccine.

A useful feature of the chosen algorithm for our prediction tasks is the ability to tune the penalty factor for a desired set of variables, from a very stringent penalty (L1, or the “lasso” penalty) to a less sparse solution (L2 quadratic or “ridge” penalty) [43], [44]. To test whether a decreasing effect of age on predicting H1N1 cPost titer altered the selection of peptide predictors, we increased the penalty for the effect of age during cross-validation, which favored the contribution of peptides over age to the resulting model [26]. Increasing the penalty factor on age, our multivariate regression model incorporated reactivity to additional peptides and retained an accuracy rate of 84.7% as assessed through LOO cross-validation (**Fig. 7c**). Intriguingly, pre-vaccine reactivity to several sets of adjacent peptides was included in the H1N1 strain neutralization outcome prediction model when the contribution of age was diminished (**Fig. 7c**). The fact that clusters of adjacent peptides (both by sequence and within the three-dimensional structure of HA) were included in these models highlights potential HA epitopes important for neutralizing H1N1 influenza.

## Discussion

Here we show that influenza whole-protein and peptide arrays revealed HA reactivity that correlated significantly with age and neutralization titer to H1N1 and B strains included in the 2008/2009 seasonal influenza vaccine. Reactivity to individual peptides correlated both positively and negatively with age or cPost titer. We indentified specific peptide epitopes that were inversely correlated with regard to age and neutralization titer of H1N1 and B strains. This result demonstrates the usefulness of the peptide array platform in identifying specific regions of HA that may be inappropriately or ineffectively targeted by the immune response in elderly individuals upon vaccination, which may contribute to the known decreased effectiveness of vaccination in older subjects. Effective predictive assays for immune response to influenza vaccine remain elusive [45]. Our ability to build accurate prediction models of neutralization titer outcome using HA peptide array reactivity suggests the utility of the platform as a diagnostic tool in the clinical setting to make meaningful observations concerning an individual’s potential to respond to influenza vaccination. Our analysis predicting virus neutralization using only age and pre-vaccine reactivity for H1N1 and H3N2 strains of influenza is of particular importance, as it implicates specific linear HA epitopes associated with a preexisting immune response to influenza that may control an individual’s ability to mount a novel, protective response upon influenza vaccination. While the current gold-standard assays for measurement of antibody responses to influenza vaccination, HAI and microneutralization, have the advantages of ubiquity and rapid adaptability to novel strains of influenza, there are several drawbacks associated with these assays. These include difficult standardization strategies, requirement of relatively high amounts of biological material, reliance on cells/cell lines, and complex tissue culture methodologies [10]. With our array-based platform we are able to directly measure specific antibody-antigen interactions with no requirement for cells or cell lines. Accordingly, standardization of a clinical assay based on peptide and/or protein reactivity could be achieved at a scale that is prohibitive using current strategies.

Our validation results not only show that reactivity on the arrays is specific, but that linear epitopes contained within HA array peptides represent authentic targets of antibodies raised against conformational HA proteins *in vivo*. Others have shown that non-linear, conformational epitopes are a target of human mAbs that bind to both head and stem regions of HA and display cross-reactive, neutralizing activity against multiple strains of virus [28], [46], [47]. While the peptide array platform as currently designed does not permit the identification of conformational targets, our systems-level approach to discover linear HA epitopes associated with effective virus neutralization may serve to compliment existing approaches, and in the future could be adapted to include conformational peptide targets as peptide mimetic technology continues to evolve [48].

One drawback of an array approach based on conventional fluorescence detection is that low-reactivity features may not be resolved above background fluorescence signal, which is a limitation of both detection reagents as well as light-based scanning technologies [49]. The implication for our study is that we have likely under-sampled the repertoire of biologically meaningful antibody reactivity in the context of influenza vaccination. This undersampling could explain the paucity of observed reactivity that significantly correlated with H3N2 neutralization titer, specifically H3N2 whole-protein and peptide antigens. Future generations of the influenza antigen array will include additional relevant protein and peptide epitopes, and we must also improve the range of detection of the platform to measure the binding of potentially relevant low-abundance and lower-affinity antibodies. Our group has recently developed two novel peptide array technologies that offer considerable improvement upon the state-of-the-art. One approach is plasmonic gold enhancement of near-infrared fluorescence, which enhances the dynamic range of array-based detection of antibody/antigen interaction by up to 100-fold [50]. We have also developed a silicon-based peptide array technology that will ultimately allow for non-fluorescence based detection of antibody/peptide interactions in real time, which could facilitate the integration of a peptide-based clinical assay to measure vaccine response scalable for use in diverse environments and as a tool for personalized medicine [16].

Our evolving technologies will compliment other assays that have been effective in predicting response to vaccines, including measurement of immune cell subsets, cytokine profiling and transcriptional analysis [51], [52], [53]. Eventually, these approaches could be integrated into a real-time, point-of-care assay that would, using readily accessible biological material (i.e. from a blood draw or nasal aspirate), predict an individual’s immune response to influenza vaccine with a high level of accuracy. This type of rapid test would be of great use for clinicians, who currently rely on broad guidelines published annually highlighting segments of the population at greatest risk [54].

In summary, we demonstrate here that a systems-level proteomics approach to measurement of the antibody responses to influenza vaccination can both identify specific antibody reactivity associated with effective neutralization of virus replication and uncover potentially important underlying mechanistic aspects of antibody-based immunity.

## Supporting Information

Figure S1 Influenza peptide array methods. Streptavidin-coated slides (Arrayit Corporation, Sunnyvale, CA) were printed in duplicate (two arrays per slide) with overlapping 19-mer peptides spanning the head region of influenza hemagglutinin (HA) and blocked with biotin. Blocked arrays were incubated in diluted patient serum, and serum antibodies that bound to cognate array features were detected with goat anti-human IgG/IgM secondary antibody conjugated to Cy3 (Jackson ImmunoResearch, West Grove, PA). Reactive features were visualized using an Axon digital scanner and analyzed with GenePix Pro 6.0 software (Molecular Devices, Sunnyvale CA).
(TIF)

Figure S2 Quantification of HA and FLAG array reactivity and validation by ELISA. (**a**) Histogram displaying median fluorescence intensity (MFI) of peptide features depicted in Figure 1a as well as FLAG peptide on an array incubated with anti-FLAG tag Ab (M2 clone, Sigma). (**b**) Graph displaying binding of HA tag Ab and FLAG tag Ab to indicated peptide targets as detected by enzyme-linked immunosorbent assay (ELISA).
(TIF)

Figure S3 Influenza array peptide reactivity correlated with age and cPost neutralization titer. Sequences corresponding to H1N1, H3N2 and B-strain influenza HA array peptides in black text. Highlighted region (orange background) corresponds to HA region containing amino acid residues that comprise the sialic acid binding domain. Below, red and blue colored peptide sequences representing peptide reactivity that was positively (red) or negatively (blue) correlated with age or cPost neutralization titer for each strain (*q* value <0.2).
(TIF)

Table S1 **Influenza array antigens.**
(TIF)

Table S2 **Donor, donor age, and corrected post-vaccine neutralization titer.**
(TIF)

Table S3 **Peptide reactivity association with age.**
(TIF)

Table S4 **Peptide reactivity association with H1N1 cPost.**
(TIF)

Table S5 **Peptide reactivity association with H3N2 cPost.**
(TIF)

Table S6 **Peptide reactivity association with B cPost.**
(TIF)
