# Supplementary figures and images for: Characterization of Influenza Vaccine Immunogenicity Using Influenza Antigen Microarrays
Source: PLoS One. 2013 May 29;8(5):e64555. doi: 10.1371/journal.pone.0064555 (PMC3667171; doi:10.1371/journal.pone.0064555)

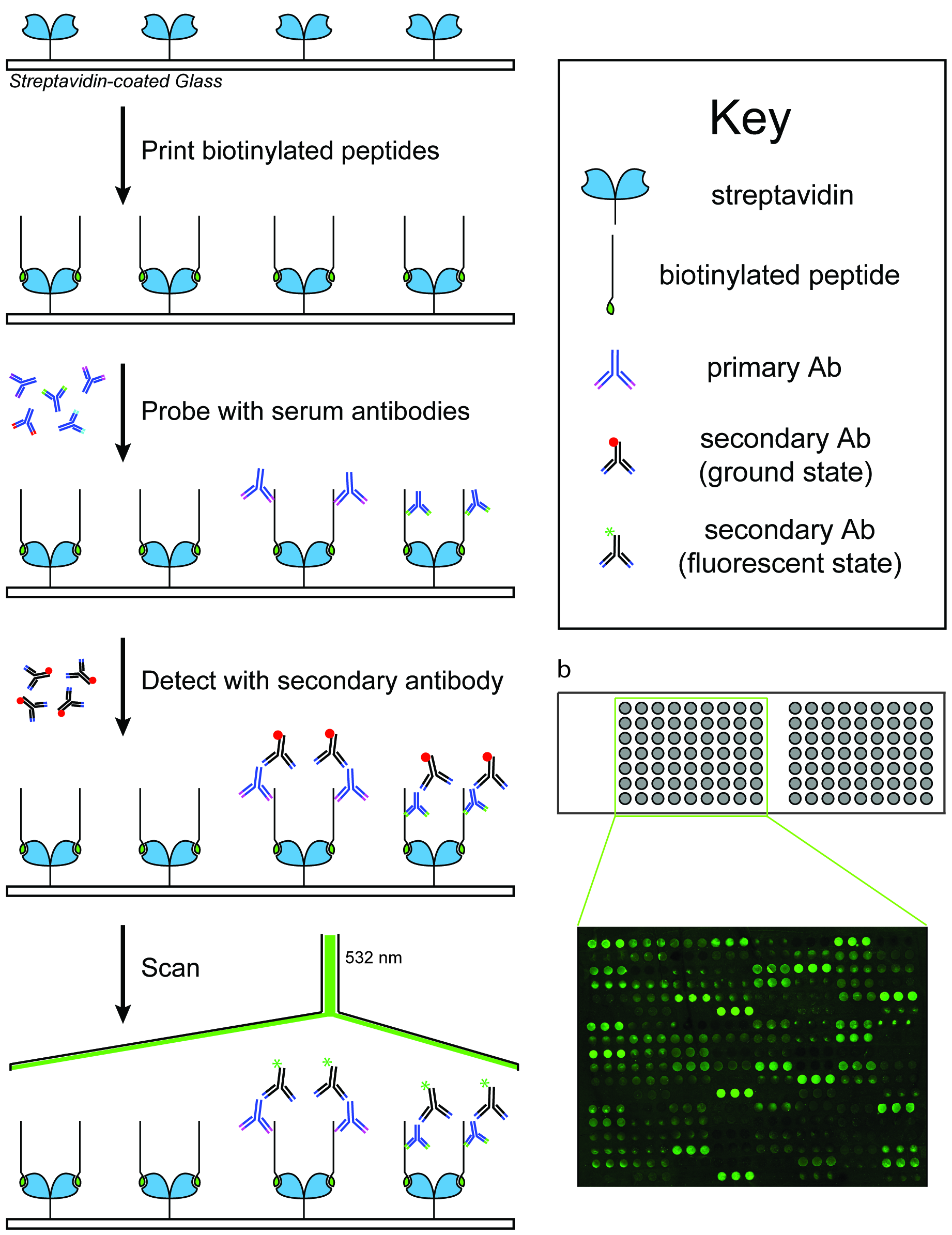

Supplement: Figure S1 — Influenza peptide array methods. Streptavidin-coated slides (Arrayit Corporation, Sunnyvale, CA) were printed in duplicate (two arrays per slide) with overlapping 19-mer peptides spanning the head region of influenza hemagglutinin (HA) and blocked with biotin. Blocked arrays were incubated in diluted patient serum, and serum antibodies that bound to cognate array features were detected with goat anti-human IgG/IgM secondary antibody conjugated to Cy3 (Jackson ImmunoResearch, West Grove, PA). Reactive features were visualized using an Axon digital scanner and analyzed with GenePix Pro 6.0 software (Molecular Devices, Sunnyvale CA). (TIF) [file pone.0064555.s001.tif]

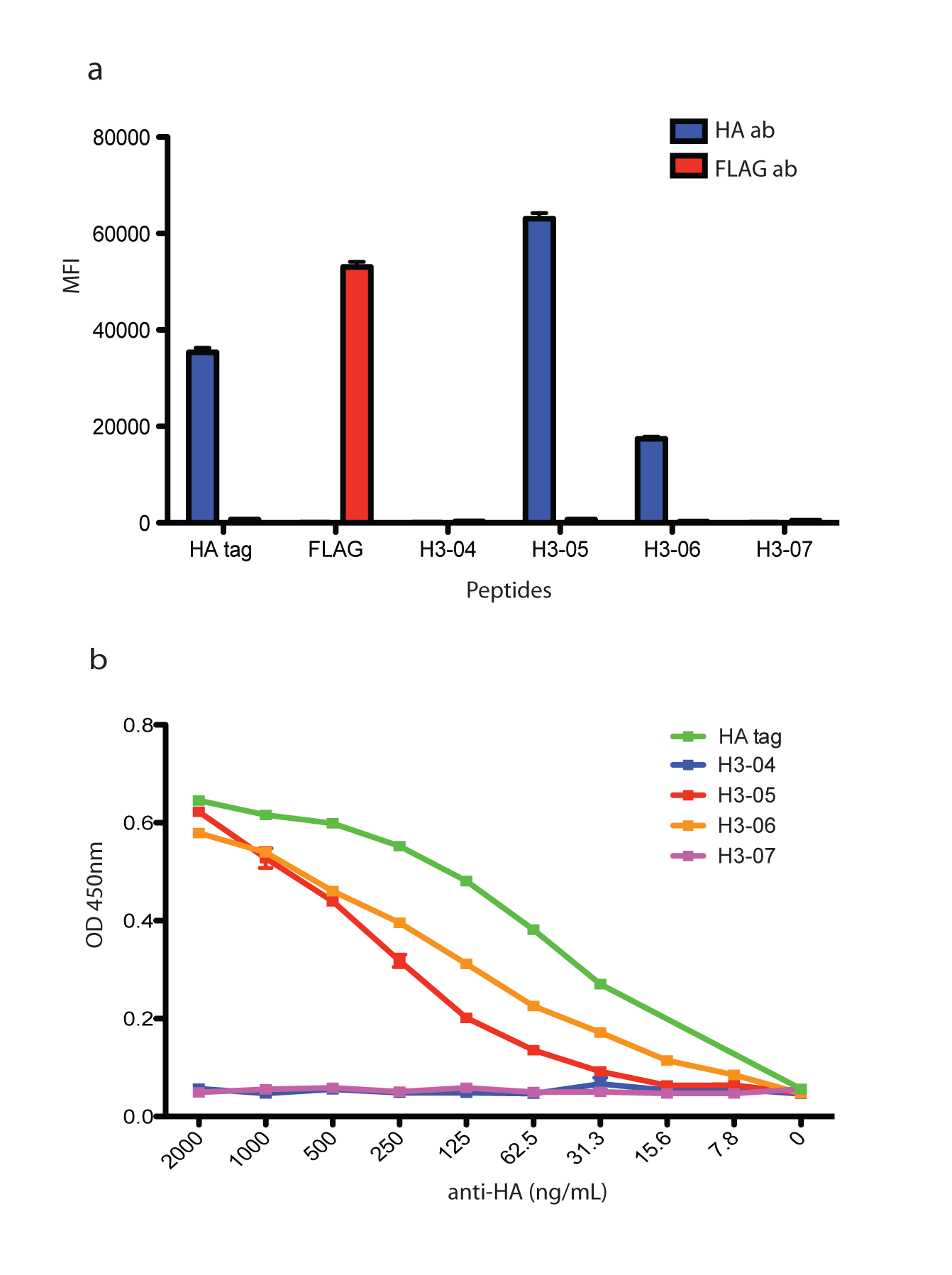

Supplement: Figure S2 — Quantification of HA and FLAG array reactivity and validation by ELISA. (a) Histogram displaying median fluorescence intensity (MFI) of peptide features depicted in Figure 1a as well as FLAG peptide on an array incubated with anti-FLAG tag Ab (M2 clone, Sigma). (b) Graph displaying binding of HA tag Ab and FLAG tag Ab to indicated peptide targets as detected by enzyme-linked immunosorbent assay (ELISA). (TIF) [file pone.0064555.s002.tif]

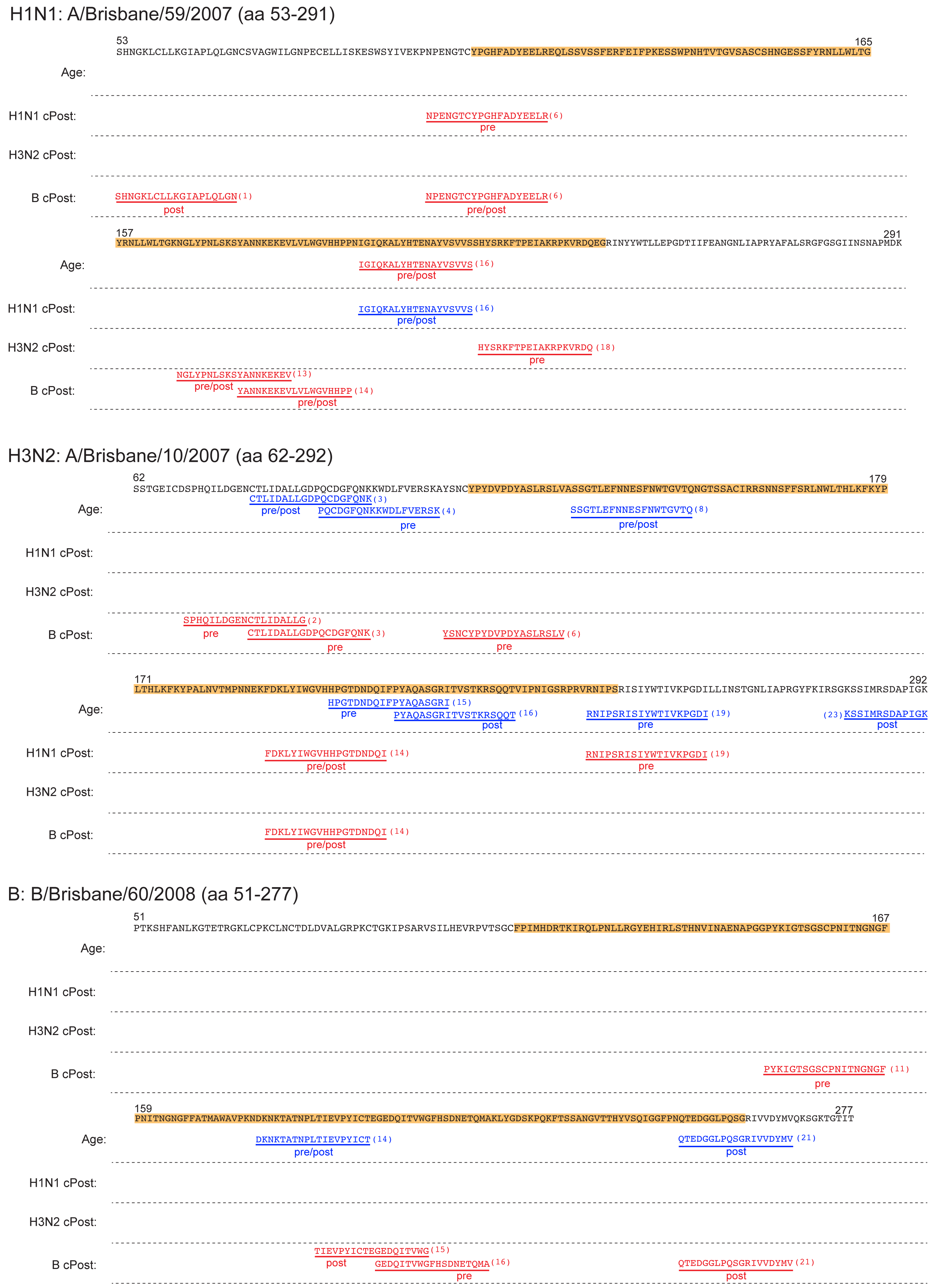

Supplement: Figure S3 — Influenza array peptide reactivity correlated with age and cPost neutralization titer. Sequences corresponding to H1N1, H3N2 and B-strain influenza HA array peptides in black text. Highlighted region (orange background) corresponds to HA region containing amino acid residues that comprise the sialic acid binding domain. Below, red and blue colored peptide sequences representing peptide reactivity that was positively (red) or negatively (blue) correlated with age or cPost neutralization titer for each strain (q value <0.2). (TIF) [file pone.0064555.s003.tif]

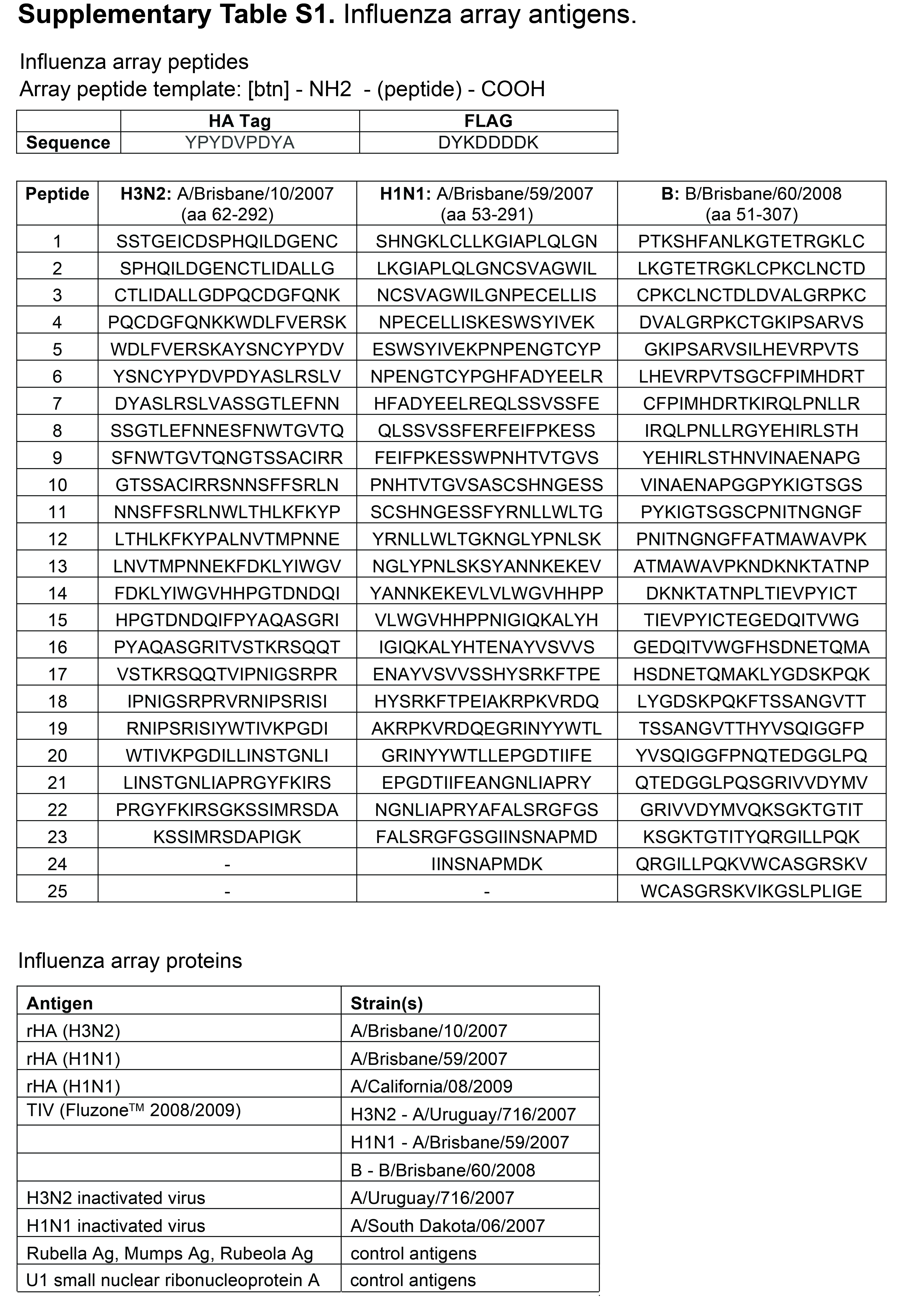

Supplement: Table S1 — Influenza array antigens. (TIF) [file pone.0064555.s004.tif]

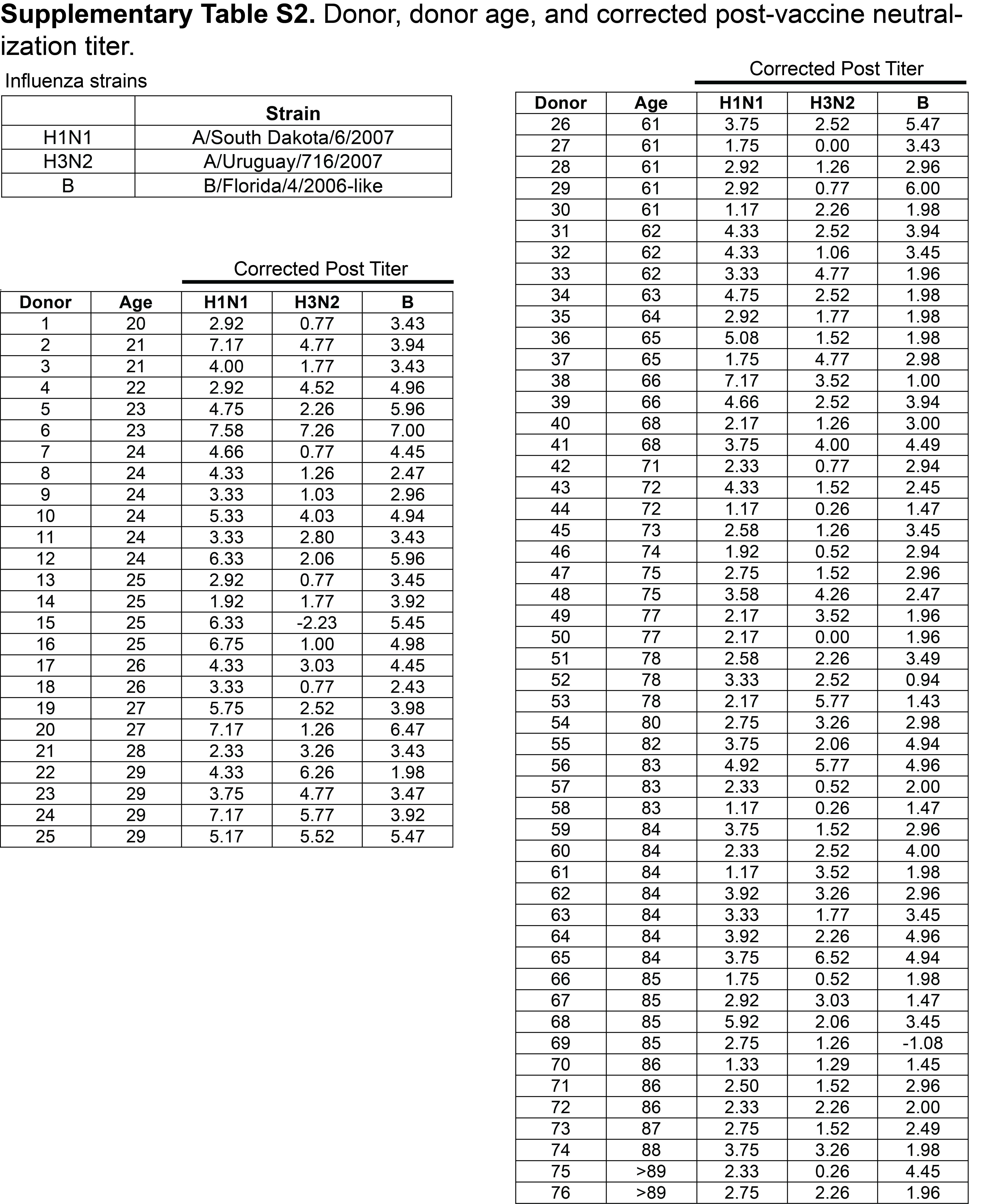

Supplement: Table S2 — Donor, donor age, and corrected post-vaccine neutralization titer. (TIF) [file pone.0064555.s005.tif]
